# Case of Acute Articular Rheumatism

**Published:** 1880-04

**Authors:** G. H. Berns

**Affiliations:** Consulting Surgeon to Hospital Department Columbia Veterinary College; 75 Adams Street, Brooklyn, N. Y.


### ABSTRACT AND SUMMARY.

### CASE DEPARTMENT.

### I.—CASE OF ACUTE ARTICULAR RHEUMATISM.

By G. H. BERNS, D. V. S.,

Consulting Surgeon to Hospital Department Columbia Veterinary College.

On Thursday, November 14, 1879, about 4 P. M., a bay gelding, from 12 to 14 years old, and in good condition, the property of Messrs. Hetfield & Ducker, of Fulton street, Brooklyn, was brought to my Infirmary, suffering, as the owners thought, from flatulent colic.

The animal was very restless, pawing and stamping with his fore feet constantly, temperature but slightly elevated, respirations and pulsations increased in frequency, visible mucous membranes injected, pupils somewhat dilated.

Examination per rectum revealed a distended condition of the bladder, and as gentle pressure upon that organ did not cause the animal to void his urine, the catheter was employed, and a considerable quantity of a rather light-colored urine was drawn off.

Although the diagnostic symptoms of colic were absent, I concluded that there might be some abdominal irritation, and an anodyne and laxative drench was administered, composed as follows:

R., Tr. Opii. Aether Sulphuric one oz.; Olei Lini Oss.

Enemas of warm water were ordered, and patient sent home, with instructions to repeat the above drench in an hour, if the symptoms continued.

Saw my patient again at 7 o'clock; found him still pawing and neighing when his attendant approached; ears pricked; pupils fully dilated; temperature, 105. Respirations and pulse very much accelerated, conjunctiva and Schneidlerian membrane quite vascular. There were no kicking at the belly, no anxious looks to the flanks, no attempts to lay down, no passing of small quantities of fæces; in short, none of the characteristic symptoms of colic.

Administered 2 grs. of sulphate of morphia, and ordered Tr. aconite in 10 drops, and Tr. opium in 2 drachm doses, to be given every hour during the night.

Saw patient again at 9 A. M. the following day, and was told that he had been quiet all night; had drank a pail of water and eaten a few carrots.

The conjunctiva was still more vascular. Respirations, 40; pulse, 95, strong and corded; temp., 105. All the symptoms of excitement had disappeared; the pupils had become somewhat contracted (probably from the effects of the opium); he presented a sleepy look, and when he was led from his box, I noticed lameness of the nigh hind leg. I called the attendant's attention to the fact, but was assured that the horse always came out stiff on that leg after a night's rest.

Not having arrived at any certain diagnosis, I concluded to treat the animal symptomatically, and continued the administration of the aconite.

On Saturday morning I saw the patient again, and found him standing in his box; learned from the attendant that he had been quiet all the previous day, had eaten a few carrots and drank freely of cold water.

When he was brought out of his box I noticed that it required the greatest exertion on his part to move his limbs, and at every step he groaned with pain. He moved with a sort of a hop and catch, similar to a horse suffering from acute laminitis of the hind feet.

All the joints were swollen, hot, and very sensitive to the touch.

The feet were no warmer than usual, and there was no throbbing of the plantar arteries.

Temp., 104; respirations and pulse, when at rest, less frequent than the morning previous.

DIAGNOSIS : *Acute Articular Rheumatism.*—A cathartic pill was given, and sodium salicylate in three drachm doses ordered in his drinking-water every four hours. Dry bed and warm clothing recommended. At 5 P. M. same day I was called to see the patient again, and found that all the symptoms of nervous excitement of Thursday afternoon had returned, in a more aggravated form.

The poor brute was standing in a corner of his stall, unable to move a limb, bathed in perspiration, head and tail elevated, pupils dilated, and on hearing the voice of his attendant he turned his head and neighed.

The swelling of the joints had somewhat subsided, but they still remained very painful to the touch.

Administered—R., potassa bromrid. ½ oz.; extr. bellad. 1 drm. per mouth; two grs. of sulph. of morphia subcutaneously, and ordered the salicylate of sodium powders continued.

On Sunday morning found the patient down; resp., 55; pulse, 100, and less forcible; temp, 104; sweating freely. On being urged and stimulated by the use of the whip, he leaped upon his feet, trembled violently, and for several minutes seemed almost unable to keep up, lifting his feet alternately, and showed symptoms of great pain. I evacuated his rectum and bladder; fæces apparently normal, urine remarkably clear and light in color, considering the high fever he had been suffering from.

Administered more morphine hypodermically, and ordered the salicylate of soda continued. In about fifteen minutes he threw himself down, almost without bending a joint.

He remained down until I saw him again at 7 P. M., when we again succeeded in getting him up. Pulse, 120, hardly perceptible; respiration, 80; temp., 104. He trembled, and showed signs of increased agony. In five minutes he threw himself down in the same manner as he had done in the morning; more morphine was injected, and sod. salicylate ordered continued. Attendant left stables at 10 P. M. on Sunday night, and found him dead the next morning.

On Monday afternoon Drs. Houseman, Mustoe and myself made a post-mortem examination, and the following pathological changes were detected:

Left kidney slightly congested; signs of hyperaemia in different portions of the large intestines; small intestines, stomach, spleen and pancreas apparently healthy; liver in parts degenerated.

The diaphragm was next removed, and the trachea, œsophagus, and large blood-vessels being severed, the lungs and heart were removed intact. The bases of both lungs were very much congested; about 20 fluid ounces of port-wine-colored fluid was found in the pericardial cavity. The pericardium showed signs of recent inflammation; the left ventricle appeared somewhat larger than usual; the endocardium, the valves and chordae-tendinæ, all showed well marked signs of inflammation, being streaked with extravasations, and opaque or dull. The left ventricle was empty, right ventricle contained a small clot of coagulated blood.

Owing to lack of time, especially on the part of the gentlemen with me, the autopsy was discontinued at this stage. Neither the joints nor the central nervous organs were examined. The non-examination of the latter I particularly regret, as I had reason to suspect what authors designate as a rheumatic metastasis to the meninges to have taken place at one period of the animal's illness.

75 ADAMS STREET, BROOKLYN, N. Y.

